# Spatiotemporal Dynamics of Suitable Habitat for *Weigela florida*

**DOI:** 10.3390/plants15121763

**Published:** 2026-06-07

**Authors:** Sixiang Zhang, Feiteng Hao, Haonan Sun, Wenpan Dong, Kangjia Liu, Yiheng Wang

**Affiliations:** 1 School of Ecology and Nature Conservation, Beijing Forestry University, Beijing 100083, China; zsx230701109@bjfu.edu.cn (S.Z.); haofeiteng@bjfu.edu.cn (F.H.); asier0016@gmail.com (H.S.); wpdong@bjfu.edu.cn (W.D.); 2 School of Landscape Architecture, Beijing Forestry University, Beijing 100083, China; 3 State Key Laboratory for Quality Ensurance and Sustainable Use of Dao-di Herbs, National Resource Center for Chinese Materia Medica, China Academy of Chinese Medical Sciences, Beijing 100700, China

**Keywords:** *Weigela florida*, ecological niche model (ENM), climate change, habitat evolution, glacial refugia

## Abstract

Global climate change profoundly impacts the geographical distribution patterns and evolutionary dynamics of plants. As a vital ornamental and ecological shrub native to the temperate regions of the Northern Hemisphere, the wild germplasm resources of *Weigela florida* are facing dual threats from habitat fragmentation and climate warming. To elucidate the biogeographical mechanisms underlying the species’ responses to climate change and to formulate scientific conservation strategies, this study simulated the spatiotemporal dynamics of suitable habitats for *W. florida* across key historical periods spanning the Late Pliocene (~3.3 million years ago), Quaternary (~2.58 million years ago), the current period, and future climate scenarios using an optimized Maximum Entropy ecological niche model, and further tracked the migration trajectories of its spatial centroids. The results indicate that precipitation conditions, dry-season temperatures, and temperature seasonality are the dominant environmental factors limiting the distribution of wild *W. florida*. During the glacial–interglacial cycles, the area of its suitable habitat fluctuated significantly. Notably, the Korean Peninsula and the southern part of Northeast China maintained high habitat suitability across all geological historical periods, serving as long-term stable Quaternary glacial refugia for the species. Under various future climate scenarios, the total suitable habitat area of *W. florida* generally exhibits a shrinking trend, with habitat loss primarily concentrated at the western and southern edges of its distribution range. In the future, its spatial centroid shows a significant tendency to migrate towards higher latitudes (northeastward) to track suitable climatic niches. This study clarifies the macroscopic driving mechanisms behind the habitat dynamics of wild *W. florida*, providing critical spatial planning guidance for the refined evaluation and long-term sustainable utilization of its germplasm resources.

## 1. Introduction

The Quaternary glacial–interglacial cycles have profoundly shaped the geographical distribution patterns, demographic histories, and spatial structures of genetic diversity of temperate woody plants in the Northern Hemisphere [1,2]. Over the long geological history, severe climatic oscillations forced numerous temperate plant taxa to retreat to lower latitudes or elevations in search of glacial refugia, and subsequently expand and recolonize higher latitudes or elevations as the climate warmed during interglacial periods [3,4]. However, since the onset of the Anthropocene, unprecedented climate warming has disrupted the natural evolutionary and range-shifting rates of species [5,6]. Rapid climate change is drastically reshaping global vegetation spatial patterns, putting the current suitable habitats of many species at severe risk of contraction, fragmentation, or even complete loss [7,8]. Therefore, under the background of global climate change, elucidating the historical spatial distribution trajectories of plants and predicting their future evolutionary trends are of tremendous theoretical and practical significance for deeply understanding the biogeographical mechanisms of species’ responses to climate change and for formulating scientific germplasm conservation strategies [9,10].

**Figure 1:**
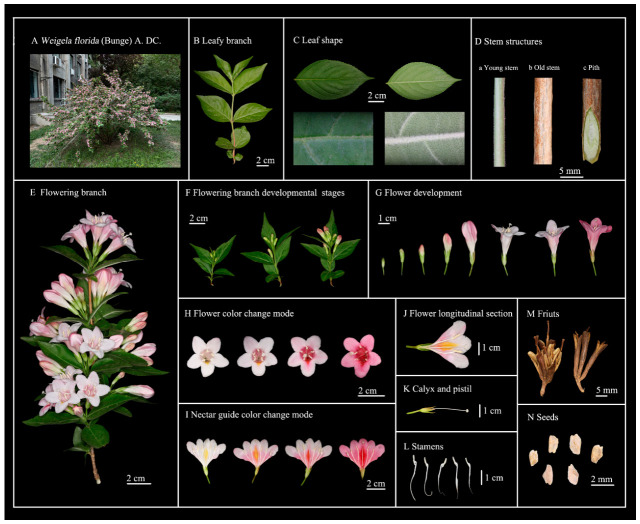
Morphology of *Weigela florida*.

*Weigela florida* (Bunge) A. DC. (Caprifoliaceae) is a typical deciduous shrub widely distributed across the temperate forest and shrubland ecosystems of East Asia, particularly spanning northeastern and northern China, the Korean Peninsula, and parts of Japan [11]. Characterized by its vibrant floral display (Figure 1), long blooming period, and robust ecological adaptability, it possesses high horticultural value and serves as an indispensable wild germplasm reservoir for modern landscape architecture and economic cultivar breeding [12,13].

Despite its significant ecological and economic importance, there is a looming and urgent need for the conservation of its wild germplasm resources, which serves as the primary motivation for this study. While *W. florida* is extensively cultivated in urban landscapes, its wild natural populations, especially the marginal and fragmented ones, are currently enduring severe threats from accelerating anthropogenic pressures, rapid urbanization, and agricultural land conversion [14]. This escalating habitat fragmentation has led to severe population isolation, impeding natural gene flow and threatening the species’ long-term evolutionary potential and genetic integrity. Crucially, as a perennial species, its reproductive and recruitment dynamics are tightly synchronized with the seasonal variations of the East Asian monsoon climate [15]. Understanding its biology and deep-time range dynamics under past climate oscillations is therefore essential for correctly interpreting how its modern populations survived past bottlenecks and how they might respond to future anthropogenic warming [1,2,16]. To date, research on *W. florida* has predominantly focused on cultivation and breeding, physiological and biochemical characteristics, and genetic diversity assessments of local populations [13,17,18]. Macro-scale studies exploring how climate change drives the large-scale geographical distribution patterns of this species remain scarce. This lack of macroscopic spatial geographic data severely limits the precise evaluation and effective conservation of wild *W. florida* germplasm resources.

Ecological Niche Models (ENMs), which establish statistical associations between known species occurrence records and environmental variables, can accurately map the potential suitable space of a species [19,20]. They have now become core analytical tools in the fields of macroecology, biogeography, and conservation biology. By integrating paleoclimatic data and future climate scenarios, ENMs can not only effectively trace the locations of spatial refugia during key geological historical periods such as the Last Glacial Maximum (LGM), but also prospectively predict potential distribution dynamics under different future Shared Socioeconomic Pathways (SSPs). Furthermore, tracking the spatiotemporal migration trajectories of the spatial centroids of a species’ suitable habitat can intuitively and quantitatively reveal the rate and direction of distribution shifts in response to climate change, thereby providing a solid scientific basis for formulating forward-looking conservation interventions [21,22].

This study focuses on wild *W. florida* and centers on the core theme of the spatiotemporal dynamics of suitable habitats. From the perspective of germplasm resource conservation, this study aims to systematically elucidate the profound impacts of climate change on its geographical distribution. This study seeks to address the following four core objectives: (1) evaluate the potential suitable habitat range of wild *W. florida* under current climate conditions and identify the dominant environmental factors limiting its distribution; (2) trace the range shift dynamics and potential glacial refugia of the species during key historical periods such as the LGM; (3) predict the expansion, contraction trends, and spatial reorganization patterns of its suitable habitat under various future climate change scenarios; and (4) quantify the migration trajectories of its suitable habitat spatial centroids. The findings will provide an important new case study for revealing the biogeographical mechanisms of temperate woody plants in the Northern Hemisphere in response to climate change.

## 2. Results

### 2.1. Evaluation of Model Accuracy and Screening of Dominant Environmental Variables

Validated by 10 Bootstrap resamplings, the average training Area Under the Receiver Operating Characteristic Curve (AUC) of the MaxEnt model reached 0.966 (Figures S1 and S2), indicating that the model possesses extremely high predictive accuracy and can be reliably used for simulating and predicting the suitable habitats of *W. florida*. Furthermore, the comparative multi-model evaluation via biomod2 revealed that, with the exception of the SRE algorithm, all other 10 algorithms (including GLM, GBM, RF, and XGBOOST) achieved exceptionally high predictive accuracy, with TSS and ROC values uniformly exceeding 0.95 (Figure S3). This high degree of cross-algorithm congruence validates that the rigorously tuned MaxEnt model used in this study is highly robust, and a single-algorithm strategy is sufficient and reliable for capturing the fundamental niche of *W. florida* without introducing significant model-dependent uncertainty.

**Figure 2:**
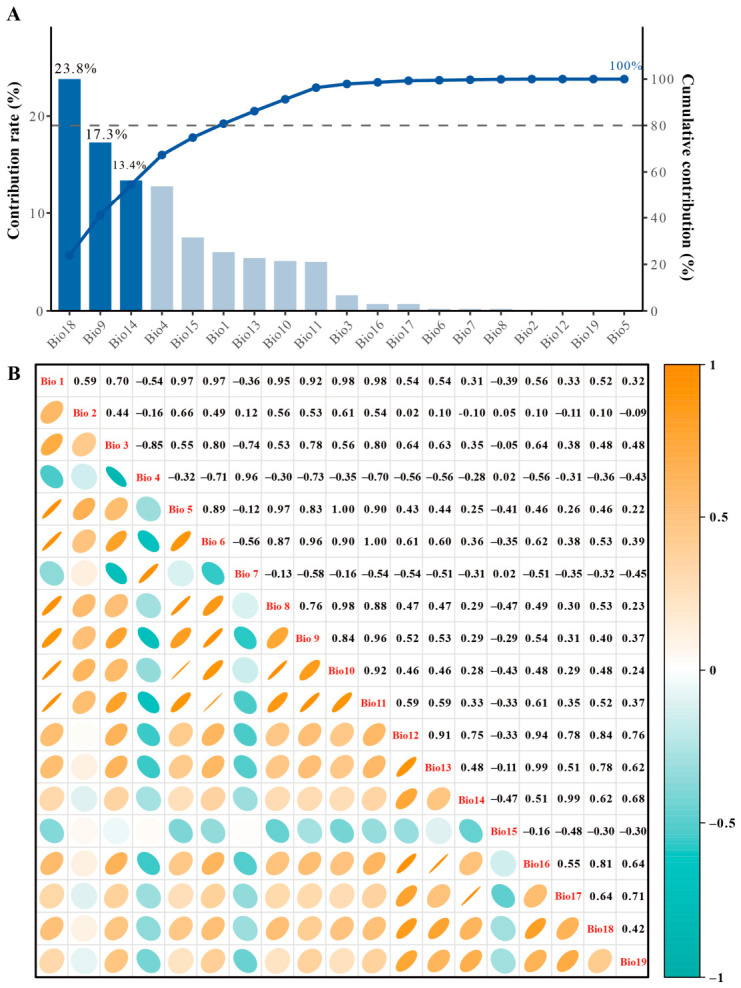
Contribution rates and correlations of bioclimatic variables. (**A**) Contribution rates and cumulative contribution rates of bioclimatic variables to the MaxEnt model. (**B**) Pearson correlation matrix among the 19 bioclimatic variables. In the Pearson correlation matrix, ellipse shape and orientation indicate the strength and direction of correlations among variables, and colors indicate positive or negative correlations. The full names and detailed descriptions corresponding to the standard bioclimatic codes (Bio1–Bio19) are provided in Table S1.

Analysis of the environmental variable contributions revealed that Precipitation of Warmest Quarter (Bio18), Mean Temperature of Driest Quarter (Bio9), and Precipitation of Driest Month (Bio14) ranked in the top three for their contributions to the model’s predictions, accounting for 23.8%, 17.3%, and 13.4%, respectively (with a cumulative contribution of 54.5%) (Figure 2A and Figure S4). In addition, Temperature Seasonality (Bio4, 12.8%) and Precipitation Seasonality (Bio15, 7.5%) were also important factors limiting the distribution. Notably, the permutation importance of Bio4 was the highest at 39.1%, highlighting the critical role of temperature seasonal fluctuations in determining the spatial pattern of *W. florida*’s suitable habitat. Combined with the Pearson correlation analysis (Figure 2B), after eliminating variables with high multicollinearity, this study ultimately retained Bio18, Bio9, Bio14, Bio4, Bio15, and Precipitation of Wettest Month (Bio13) as the core modeling variables. Overall, these six variables comprehensively reflect precipitation abundance (Bio18, Bio14, Bio13), dry-season heat (Bio9), and climatic seasonal rhythms (Bio4, Bio15), demonstrating that the geographic distribution of wild *W. florida*’s potential suitable habitat is jointly driven by water availability, dry-season temperatures, and climatic seasonal variations.

### 2.2. Distribution of Potential Suitable Habitats Under Current Climate Conditions

**Figure 3:**
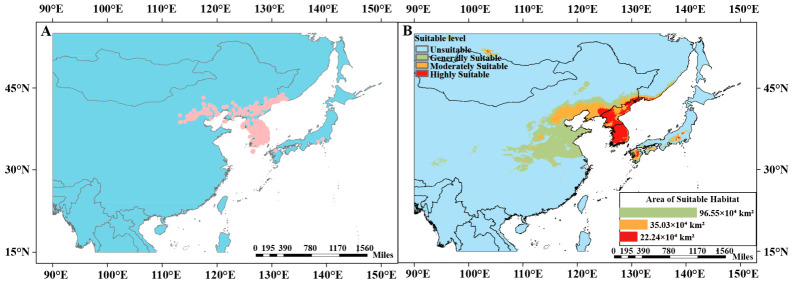
Current distribution records and potential suitable habitat of *W. florida*. (**A**) Current occurrence records of *W. florida*. (**B**) Predicted current potential suitable habitat of *W. florida* based on the MaxEnt model. Suitable habitats were classified into unsuitable, generally suitable, moderately suitable, and highly suitable areas.

Simulation results under the current climate background showed that the suitable habitats for *W. florida* are primarily concentrated in East Asia, exhibiting significant belt-like and patch-like mosaic distribution characteristics spatially (Figure 3). The total area of the current suitable habitat is approximately 153.83 × 10^4^ km^2^. Among them, the low-suitability area is the most widely distributed (96.55 × 10^4^ km^2^, accounting for 62.77% of the total suitable area), mainly covering Northeast China, North China, the Korean Peninsula, and parts of Japan. The high-suitability area shows obvious geographical clustering (22.24 × 10^4^ km^2^, accounting for 14.46%), largely confined to the Korean Peninsula and its adjacent core regions. The moderate-suitability area (35.03 × 10^4^ km^2^, accounting for 22.77%) is mostly distributed in a ring shape around the periphery of the high-suitability area.

### 2.3. Spatiotemporal Dynamics of Suitable Habitats Under Historical Climate Conditions

**Figure 4:**
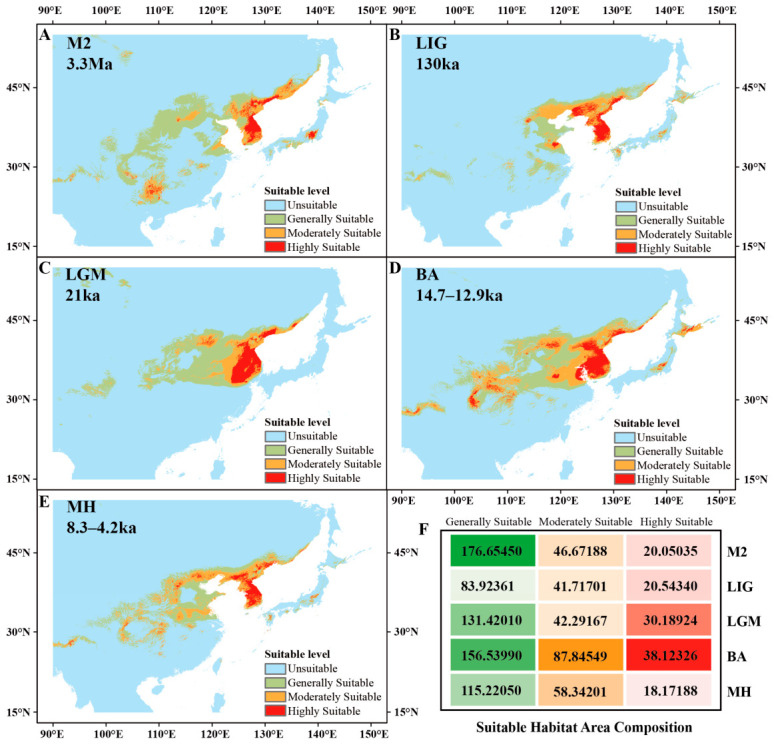
Potential suitable habitat distribution and area composition of *W. florida* during historical periods. (**A**–**E**) Potential suitable habitat distribution of *W. florida* during the M2 (Marine Isotope Stage M2, ~3.3 Ma), LIG (Last Interglacial, ~130 ka), LGM (Last Glacial Maximum, ~21 ka), BA (Bølling–Allerød, 14.7–12.9 ka), and MH (Mid-Holocene, 8.3–4.2 ka) periods, respectively. (**F**) Area composition of generally suitable, moderately suitable, and highly suitable habitats during different historical periods. heatmap colors indicate the relative magnitude of suitable habitat area within each suitability category, with darker colors representing larger areas.

Throughout various geological historical periods, the suitable habitats of *W. florida* have consistently been centered in East Asia, but their spatial coverage and suitability level structure have undergone significant dynamic fluctuations. Overall, with the exception of the Last Interglacial (LIG), the total suitable habitat areas in all other historical periods were generally larger than the current level (Figure 4).

Specifically, during the LIG, the suitable habitat contracted severely, with the total area dropping to 146.18 × 10^4^ km^2^, and the areas of all suitability levels were at historical lows, indicating that the high-temperature climate during this period strongly restricted the habitat of *W. florida*. Entering the Last Glacial Maximum (LGM), the total area expanded slightly, recovering to 203.90 × 10^4^ km^2^. During the M2 period, the suitable habitat range further expanded (243.38 × 10^4^ km^2^); by the Bølling–Allerød (BA) warm period, the total area reached its historical peak (282.51 × 10^4^ km^2^), and the areas of low-suitability (156.54 × 10^4^ km^2^), moderate-suitability (87.85 × 10^4^ km^2^), and high-suitability (38.12 × 10^4^ km^2^) habitats were all at their highest levels, reflecting that the climatic conditions during this stage were highly aligned with the species’ ecological requirements. By the Mid-Holocene (MH), the total area fell back to 191.73 × 10^4^ km^2^; although reduced compared to the BA period, it remained markedly larger than the habitat scale under contemporary climate conditions.

### 2.4. Predictions of Suitable Habitats Under Future Climate Scenarios

**Figure 5:**
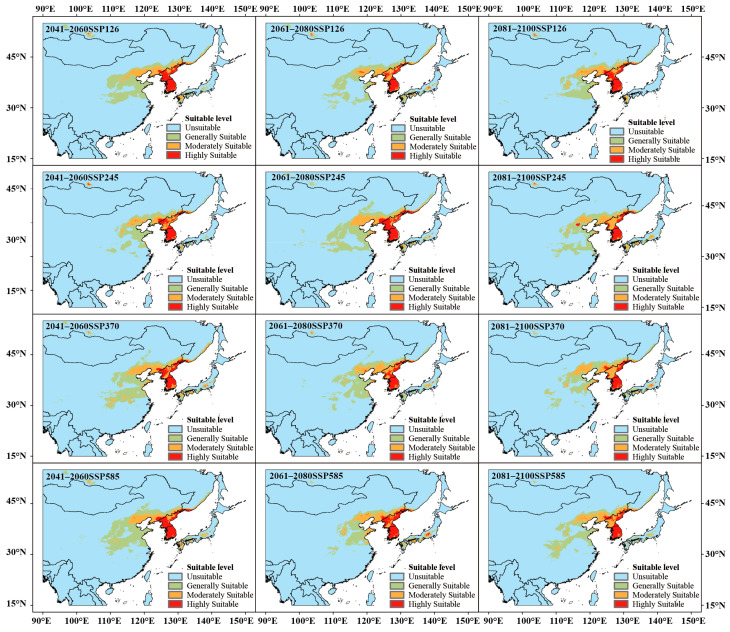
Potential suitable habitat distribution of *W. florida* under future climate scenarios. The figure shows the spatial distribution of potential suitable habitats of *W. florida* during three future periods, 2041–2060, 2061–2080, and 2081–2100, under four climate scenarios: SSP126, SSP245, SSP370, and SSP585. Suitable habitats were classified into unsuitable, generally suitable, moderately suitable, and highly suitable areas.

Under four future greenhouse gas emission scenarios (SSPs), the overall spatial pattern of *W. florida*’s suitable habitat is similar to the current one, with the high-suitability areas still anchored in southern Northeast China, the Korean Peninsula, and parts of Japan. Future habitat changes will primarily occur at its distribution edges, especially in the western, southern, and northern transitional zones, exhibiting significant scenario and temporal heterogeneity (Figure 5). In terms of the overall trend, across most predicted scenarios and time points, the future total suitable habitat area will face a reduction, although transient increases in suitable climate space may occur in the mid-century under certain scenarios (Figure 5).

Under the SSP1-2.6 scenario, the total suitable habitat area shows a continuous shrinking trend over time, monotonically decreasing from the current 153.83 × 10^4^ km^2^ to 117.89 × 10^4^ km^2^ in the 2090s. The SSP2-4.5 scenario presents a fluctuation of “contraction–rebound–contraction” (dropping to 110.27 × 10^4^ km^2^ in the 2050s, rebounding to 155.44 × 10^4^ km^2^ in the 2070s, and falling again to 125.13 × 10^4^ km^2^ in the 2090s).

Under the SSP3-7.0 scenario, after a brief, slight expansion in the 2050s (159.42 × 10^4^ km^2^), it rapidly declines, dropping to 122.35 × 10^4^ km^2^ by the 2090s. The extreme SSP5-8.5 scenario similarly exhibits a drastic fluctuation of increasing then decreasing, reaching 169.05 × 10^4^ km^2^ in the 2050s before sharply declining to 121.40 × 10^4^ km^2^ in the 2070s.

Regarding habitat quality, the low-suitability area consistently dominates and profoundly influences the trajectory of the total area’s increase or decrease. In contrast, the high-suitability area does not show any sustained expansion trend, fluctuating between 15.98 × 10^4^ km^2^ and 25.38 × 10^4^ km^2^ (representing a variation of 10.39% to 16.50% relative to the current total suitable area) over the long term.

### 2.5. Spatial Transformation Patterns of Future Suitable Habitats: Expansion, Contraction, and Stability

By overlaying the current and future suitable habitat layers, it was found that *W. florida*’s habitat will simultaneously undergo expansion, contraction, and reorganization in response to future climate warming. The spatial transformation results show that across all periods and scenarios, the stable areas (overlapping suitable areas) maintain absolute dominance, with areas ranging between 91.02 × 10^4^ km^2^ and 112.31 × 10^4^ km^2^, and highly coincide spatially with the current core suitable areas. This reveals that the core populations of *W. florida* possess strong environmental and ecological resilience when facing future climate shocks (Figure S5).

**Figure 6:**
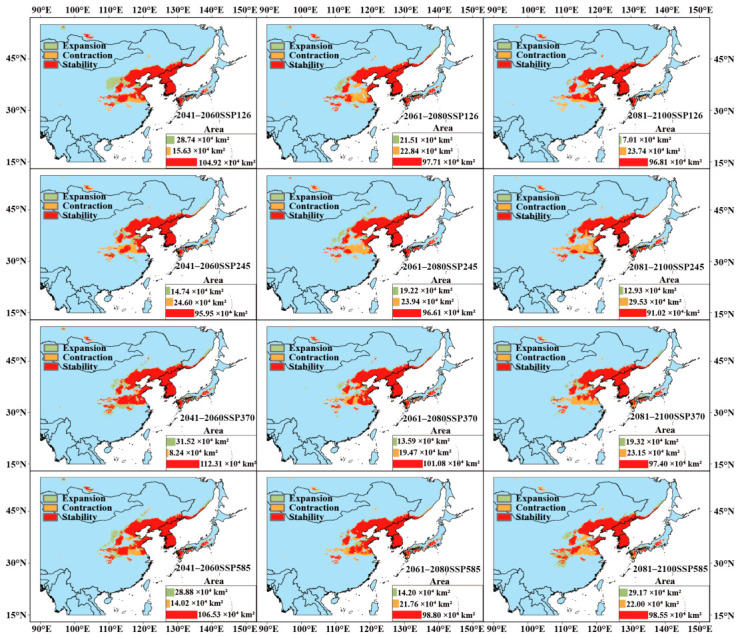
Distribution of habitat expansion, contraction, and stability areas of *W. florida* under future climate scenarios. The figure shows the spatial distribution of habitat expansion, contraction, and stability areas of *W. florida* during 2041–2060, 2061–2080, and 2081–2100 under SSP126, SSP245, SSP370, and SSP585 scenarios, relative to the current suitable habitat.

The colonization of new habitats (expansion areas, 7.01 × 10^4^ km^2^ to 31.52 × 10^4^ km^2^) mostly occurs in the higher-latitude peripheries of the existing suitable habitats (Figure 6). Conversely, habitat loss (contraction areas, 8.24 × 10^4^ km^2^ to 29.53 × 10^4^ km^2^) is primarily concentrated at the lower-latitude edges and climate-sensitive transitional zones of the suitable habitats. An analysis of the projected extremes indicates that the maximum potential expansion occurs under the SSP3-7.0 scenario during 2041–2060 (31.52 × 10^4^ km^2^), while the most severe habitat loss is predicted to occur under the SSP2-4.5 scenario during 2081–2100 (29.53 × 10^4^ km^2^).

### 2.6. Migration Trajectories of Suitable Habitat Spatial Centroids

Spatial tracking of the distribution centers (centroids) revealed the geographic response trajectories of *W. florida* under climate forcing. During historical stages, its suitable habitat centroid primarily roamed the region from eastern China to North China. From the M2 period to the present, the centroid has not exhibited a linear progression in a single direction, but rather multi-directional fluctuating shifts alongside alternating cold and warm paleoclimates. Notably, the current suitable habitat centroid has shifted significantly towards the northeast compared to certain key historical periods, corroborating the pulling effect of post-glacial climate warming on the spatial center of gravity of this species (Figure S6).

Under future climate scenarios, the baseline for centroid migration remains anchored near the current center of gravity, but different climate forcing pathways (SSPs) trigger divergent migration trajectories. Under lower emission pathways (SSP1-2.6 and SSP2-4.5), the centroid predominantly exhibits short-distance, back-and-forth oscillations within a localized range. In contrast, during the middle and late stages of high emission pathways (SSP3-7.0 and SSP5-8.5), the magnitude of centroid shift intensifies significantly (Figure S6). This centroid dynamic indicates that continuous future climate change will force phase-specific reorganizations of *W. florida*’s suitable habitat spatial patterns, and the degree of response is closely related to the intensity of greenhouse gas emissions.

## 3. Discussion

### 3.1. Driving Mechanisms of Climatic Variables on the Geographical Distribution of W. florida

The geographical distribution pattern of a species is the result of its long-term evolutionary adaptation to the environment, profoundly reflecting the response of its ecophysiological requirements to large-scale climatic conditions. Our model (AUC = 0.966) indicates that precipitation conditions (Precipitation of Warmest Quarter, Bio18; Precipitation of Driest Month, Bio14) and dry-season temperatures (Mean Temperature of Driest Quarter, Bio9) are the key factors limiting the potential suitable habitat distribution of *W. florida* (Figure 2). This is highly consistent with the niche characteristics of typical woody plants in the temperate deciduous broad-leaved forests of the Northern Hemisphere [23,24,25,26,27,28].

As a shrub primarily distributed in the East Asian monsoon region, *W. florida* requires abundant precipitation during the growing season (the warmest quarter) to maintain its vigorous vegetative growth and reproductive development [29]. In contrast, during the arid winter and early spring, suitable temperatures and baseline precipitation can effectively reduce the physiological drought risk of overwintering buds. Furthermore, the extremely high permutation importance of Temperature Seasonality (Bio4, 39.1%) indicates that climatic seasonal rhythms play a decisive role in shaping its distribution boundaries. This confirms that temperate deciduous plants, through long-term evolution, have developed phenological rhythms that are highly synchronized with the East Asian monsoon climate [30,31]; once this seasonal rhythm is disrupted, their suitable space will be significantly compressed [32].

### 3.2. Historical Distribution Dynamics and Refugia Maintenance

Frequent climatic oscillations during the Quaternary reshaped the demographic history of East Asian plants [33,34,35]. Our simulation results reveal that the spatial dynamics of *W. florida* across different geological historical periods did not follow a simple “glacial contraction–interglacial expansion” model. Notably, during the Last Interglacial (LIG), due to global temperatures being significantly higher than present, its suitable habitat area experienced a historical trough (146.18 × 10^4^ km^2^). Conversely, during the Last Glacial Maximum (LGM) and even the colder M2 period, its suitable habitat area was actually larger than it is currently (Figure 4). This phenomenon has been reported in certain cold-tolerant temperate flora in East Asia [36], indicating that under glacial climates, along with the drop in sea level (e.g., the exposure of the East China Sea continental shelf) and the strengthening of the continental climate, *W. florida* may have found expansive suitable habitats at lower latitudes or along newly exposed land margins [37].

During the Bølling–Allerød (BA) warm period, the suitable habitat area reached its historical peak (282.51 × 10^4^ km^2^). This likely benefited from the synchronized effects of post-glacial warming and increased precipitation, which provided an optimal window for its proliferation. Although the absolute total area expanded during the LGM, possibly due to the colonization of newly exposed peripheral landmasses, these temporary maritime habitats were subsequently lost during warmer interglacial periods. Despite such drastic spatial fluctuations across the glacial–interglacial cycles, the Korean Peninsula and the southern part of Northeast China consistently maintained high habitat suitability. This persistent spatial overlap strongly suggests that these stable core regions functioned as long-term Quaternary glacial refugia for *W. florida*, ensuring the survival of its main lineages through various climate bottlenecks (such as the LIG). These refugia played an irreplaceable foundational role in maintaining the modern genetic diversity of the species [38,39,40,41].

### 3.3. Reshaping of Spatial Patterns and Centroid Migration

Since entering the Anthropocene, unprecedented climate warming has accelerated the reshaping of plant distribution patterns. This study demonstrates that under multiple future climate scenarios (SSPs), the total suitable habitat area of *W. florida* generally exhibits a shrinking trend, which aligns with the pessimistic predictions for many temperate endemic species under future climates. Although transient expansions of suitable climate niches are projected during specific periods (e.g., the 2050s under SSP3-7.0 and SSP5-8.5), in the long run, high-intensity greenhouse gas emissions will lead to the continuous degradation of its habitat (Figure 5).

Analyses of spatial transformation and centroid migration further reveal the geographic trajectories of *W. florida* in response to climate change. The future loss of suitable habitat (contraction areas) is primarily concentrated along the western and southern edges, as well as the transitional zones of its distribution range. These populations, located at the trailing edge of the distribution range, typically endure the most severe environmental stresses. As the overall centroid shifts toward higher latitudes in the northeast, *W. florida* exhibits a tendency to track suitable climatic niches toward higher latitudes. However, the limited natural migration and dispersal capacity of woody plants may lag far behind the current rate of climate warming [42,43]. This climate migration lag places marginal populations at an extremely high risk of local extinction.

### 3.4. Limitations and Uncertainties

Although the optimized MaxEnt model and the regionally customized BCC-CSM2-MR climate model provided high predictive accuracy and robust spatial insights for *W. florida*, several inherent limitations and uncertainties must be acknowledged. First, relying on a single GCM for future climate projections introduces substantial uncertainty regarding regional circulation biases and climate sensitivity envelopes. While BCC-CSM2-MR is highly optimized for the East Asian monsoon region, incorporating a multi-model ensemble averaging approach in future studies could buffer against the potential extremes or systematic errors of any single GCM, thereby providing a more comprehensive envelope of potential habitat shifts [44]. Second, while relying on a single modeling algorithm can sometimes introduce model-dependent variation, our supplementary evaluation using the biomod2 ensemble platform demonstrated that 10 different algorithms (TSS > 0.95 and ROC > 0.95) yielded highly congruent predictive performances [45]. This confirms that our rigorously tuned MaxEnt model (via the “ENMeval” package) provides a robust representation of the species’ niche, effectively mitigating algorithm-induced uncertainties.

Third, deep-time projections back to the M2 period (approximately 3.3 million years ago) rely heavily on the strict assumption of long-term phylogenetic niche conservatism [46]. From a niche conservatism perspective, assuming that a species’ ecological niche requirements have remained entirely unchanged over such an extensive geological timeframe is highly contentious. In reality, complex evolutionary forces, historical hybridization events, or localized adaptive differentiation since the Pliocene could have triggered significant niche evolution or shifts. These factors may cause the species’ actual historical distribution to deviate from our fundamental climate-envelope predictions. Therefore, the unverified validity of this niche conservatism assumption across macroevolutionary timescales represents a limitation of this deep-time modeling framework.

Finally, as a perennial deciduous shrub with a long generation time, *W. florida*’s actual distribution is heavily constrained by biological factors such as dispersal limitations, delayed recruitment, and biotic interactions. The omission of these dynamic biological parameters in correlative ENMs might result in an overestimation of the species’ migration rate, underscoring a potential ‘climate migration lag’ under rapid anthropogenic warming [43,47]. Future research integrating landscape genomics and mechanistic models will be critical to refine these predictions and guide precision conservation efforts.

## 4. Materials and Methods

### 4.1. Species Occurrence Data

The current distribution data for *W. florida* were primarily obtained from databases, such as the Global Biodiversity Information Facility (GBIF, https://www.gbif.org/, Occurrence Download, https://doi.org/10.15468/dl.wwsh94, accessed on 24 October 2025). To ensure taxonomic and spatial accuracy, the acquired occurrence points were rigorously cross-referenced and revised based on authoritative professional databases, including the Flora of China and the World Checklist of Vascular Plants (WCVP), yielding an initial set of 1054 valid records. Subsequently, spatial cleaning was performed on the data to remove records lacking precise coordinates or representing duplicated points. To eliminate sampling bias and spatial autocorrelation, which could lead to overfitting of the MaxEnt model [48], a spatial rarefaction method using a 10 km × 10 km grid was applied, retaining only one representative occurrence point per grid cell. Following these treatments, 732 independent occurrence points were ultimately retained for subsequent model construction.

### 4.2. Collection and Processing of Environmental Variables

This study utilized 19 bioclimatic variables (Table S1) representing current climate conditions (1970–2000) from the WorldClim database (https://worldclim.org/, accessed on 25 October 2025) as the baseline environmental variables, with a spatial resolution of 2.5 arc-minutes, while paleoclimatic data for three time slices (M2, LIG, and BA periods) spanning the Pleistocene and Holocene were acquired from paleoclimate databases (http://www.paleoclim.org, accessed on 25 October 2025). All data were uniformly converted to ASCII format using ArcGIS v.10.8 (Esri, Redlands, CA, USA) to meet the operational requirements of MaxEnt. Given the complex multicollinearity that frequently exists among environmental variables, a rigorous screening process was conducted based on the contribution rates of the variables to the model predictions and their correlations. First, the occurrence points and the 19 bioclimatic variables were imported into MaxEnt 3.4.4 for a pre-run to obtain the relative contribution rate of each variable (ranging from 0 to 1). Next, the Pearson correlation coefficients (*r*) among the 19 environmental variables were calculated using ENMTools v1.4.4 [49]. When a high degree of spatial collinearity (|*r*| > 0.8) was detected between a pair of variables, only the variable with the higher contribution rate to the species distribution was retained to minimize the interference of collinearity on model accuracy. Ultimately, six key environmental factors were selected for formal modeling.

### 4.3. Optimization of the MaxEnt Model

To prevent model overfitting, the “ENMeval” package in R v.4.4.2 [50] was used to systematically optimize two key parameters of the MaxEnt model: the Regularization Multiplier (RM) and the Feature Class (FC) [51]. The RM acts as a smoothing parameter that penalizes model complexity, thereby allowing for more localized predictions. The FC is used to capture the different response patterns of a species to environmental gradients. This study evaluated 10 RM settings (ranging from 0.5 to 5.0, with an increment of 0.5) and 5 parameter combinations derived from 5 basic features (Linear [L], Quadratic [Q], Product [P], Threshold [T], and Hinge [H]): L, H, LQ, LQH and LQHP. Based on the corrected Akaike Information Criterion (AICc), the complexity and goodness-of-fit of the models were comprehensively evaluated by calculating the ΔAICc values for the different parameter combinations, in conjunction with the 10% training omission rate (or.10p.avg) and the difference between training and test AUC (auc.diff.avg). Finally, the RM and FC combination corresponds to delta. AICc = 0 was selected as the optimal parameter set to construct the final model.

To validate the robustness of our single-algorithm approach and address potential model-dependent uncertainties, a comparative multi-model evaluation was additionally conducted using the biomod2 package in R [45]. We ran 11 different algorithms, including Generalized Linear Models (GLM), Generalized Boosted Models (GBM), Generalized Additive Models (GAM), Classification Tree Analysis (CTA), Surface Range Envelope (SRE), Flexible Discriminant Analysis (FDA), Multiple Adaptive Regression Splines (MARS), Random Forest (RF), MAXENT, MAXNET, and XGBOOST. The True Skill Statistic (TSS) and the area under the Receiver Operating Characteristic (ROC) curve were utilized to evaluate and compare the predictive accuracy of these individual models against our optimized MaxEnt model.

### 4.4. MaxEnt Simulation and Model Accuracy Evaluation

The filtered occurrence points and the six key climatic variables were imported into MaxEnt 3.4.1 (American Museum of Natural History, New York, NY, USA) for formal modeling. The model run employed a Bootstrap resampling approach, allocating 75% of the occurrence points for model training and the remaining 25% for model testing. The maximum number of background points was set to 10,000, and the maximum number of iterations was set to 5000. A Jackknife test was used to quantitatively assess the importance of each climatic variable to the distribution of *W. florida*. The model was replicated 10 times, and the average logistic value was adopted as the final prediction result, with all other parameters left at their default software settings. Model prediction accuracy was evaluated using the Area Under the Receiver Operating Characteristic Curve (AUC), which has a theoretical value ranging from 0 to 1. In this study, model performance was measured by the average test AUC value across the 10 replicates. Generally, an AUC > 0.9 indicates that the model’s predictive performance is extremely accurate [52].

### 4.5. Classification of Suitable Habitat Levels

The suitability index output by MaxEnt ranges from 0 to 1, with values closer to 1 indicating higher habitat suitability. This study selected the “maximum training sensitivity plus specificity logistic threshold” (MTSS) as the cutoff to distinguish between suitable and unsuitable areas, as this threshold has been widely proven to possess high objectivity and discriminability in ecological niche modeling [53]. The average MTSS value from the 10 simulation runs was taken as the final classification threshold: areas with values below this threshold were classified as unsuitable habitats. For areas above the threshold, the Reclassify tool in ArcGIS 10.8 was used to further divide the suitable habitat into three levels using the Natural Breaks (Jenks) method: low-suitability, moderate-suitability, and high-suitability areas. Finally, the spatial area of each suitability level was calculated under the Goode Homolosine projection for comparative analysis [54].

### 4.6. Simulation of Historical Suitable Habitats

For the Last Glacial Maximum (LGM) and the Mid-Holocene (MH), this study utilized paleoclimate data from the CCSM4 model downloaded from the WorldClim database, also at a spatial resolution of 2.5 arc-minutes. During the modeling process, the same six key climatic variables used for the current period were selected, and the historical climate data were imported as projection layers into the optimized MaxEnt model to simulate the historical suitable habitats. All operational parameters and suitability classification methods for this historical model were identical to those used in the current suitable habitat simulation.

### 4.7. Future Suitable Habitats and Centroid Migration

This study employed the BCC-CSM2-MR global climate model from the Coupled Model Intercomparison Project Phase 6 (CMIP6) for future scenario simulations [55]. This model, developed by the National Climate Center of China, was selected because it has been extensively demonstrated to exhibit superior performance and higher simulation accuracy for key climatic drivers specifically within the East Asian monsoon region—the core distribution range of *W. florida*—compared to many other global climate models (GCMs) [56]. It is therefore widely recommended for short-term climate predictions and climate change assessments in China and East Asia [57]. To comprehensively explore future dynamic distributions, two comparative dimensions were selected: (1) To explore the ultimate impacts under different emission intensities, climate data for the period 2081–2100 (the 2080s) under four Shared Socioeconomic Pathways (SSP1-2.6, SSP2-4.5, SSP3-7.0, and SSP5-8.5) were utilized. (2) To explore the time-series evolution under an extreme climate scenario, climate data under the SSP5-8.5 scenario were selected for three consecutive periods: 2041–2060 (the 2040s), 2061–2080 (the 2060s), and 2081–2100 (the 2080s). Based on the suitable habitat simulation results across different eras and climate scenarios, the “Mean Center” tool in ArcGIS was used to calculate and extract the spatial distribution centroids of the suitable habitats for *W. florida* in each period. This allowed for the quantification and clarification of the migration directions and spatial transformation patterns of the suitable habitats under varying future climate scenarios.

## Data Availability

The current distribution data for *Weigela florida* were primarily obtained from databases such as the Global Biodiversity Information Facility (GBIF, https://www.gbif.org/, accessed on 24 October 2025). This study utilized 19 bioclimatic variables representing current climate conditions (1970–2000) from the WorldClim database (https://worldclim.org/, accessed on 25 October 2025) as the baseline environmental variables, with a spatial resolution of 2.5 arc-minutes.

## Supplementary Materials

The following supporting information can be downloaded at: https://www.mdpi.com/article/10.3390/plants15121763/s1. Figure S1. Jackknife test of environmental variable importance for the MaxEnt model of *Weigela florida*. The turquoise bars indicate the model gain without the corresponding variable, the blue bars indicate the gain using only the corresponding variable, and the red bar indicates the gain using all variables. Figure S2. Receiver operating characteristic curve of the MaxEnt model for *Weigela florida*. The red line represents the mean ROC curve, the blue line represents ± one standard deviation, and the black diagonal line represents random prediction. The mean AUC value was 0.966, indicating high model predictive performance. Figure S3. TSS and ROC evaluations of every single model of *Weigela florida*. Figure S4. Response curves of *Weigela florida* to three key environmental variables. A: Bio9; B: Bio14; C: Bio18. The red line represents the mean response of 10 replicate MaxEnt runs, and the blue area represents ± one standard deviation. Figure S5. Changes in suitable habitat area of *Weigela florida* under historical and future climate scenarios. (A) Changes in suitable habitat area from historical periods to the current period. (B–E) Changes in suitable habitat area under SSP126, SSP245, SSP370, and SSP585 scenarios, respectively. The bars represent total suitable area, generally suitable area, moderately suitable area, and highly suitable area. Figure S6. Centroid migration trajectories of suitable habitats of *Weigela florida* under historical and future climate scenarios. (A) Centroid migration trajectory of suitable habitats from historical periods to the current period. (B–E) Centroid migration trajectories from the current period to future periods under SSP126, SSP245, SSP370, and SSP585 scenarios, respectively. Arrows indicate the direction of centroid migration. Table S1. List of the 19 bioclimatic variables from the WorldClim database used in this study.
