# Supplementary material for: Spatiotemporal Dynamics of Suitable Habitat for Weigela florida
Source: Plants (Basel). 2026 Jun 7;15(12):1763. doi: 10.3390/plants15121763 (PMC13307421; doi:10.3390/plants15121763)

Figure S1. Jackknife test of environmental variable im-portance for the MaxEnt model of *Weigela florida*. The turquoise bars indicate the model gain without the corresponding variable, the blue bars indicate the gain using only the corresponding variable, and the red bar indicates the gain using all variables.

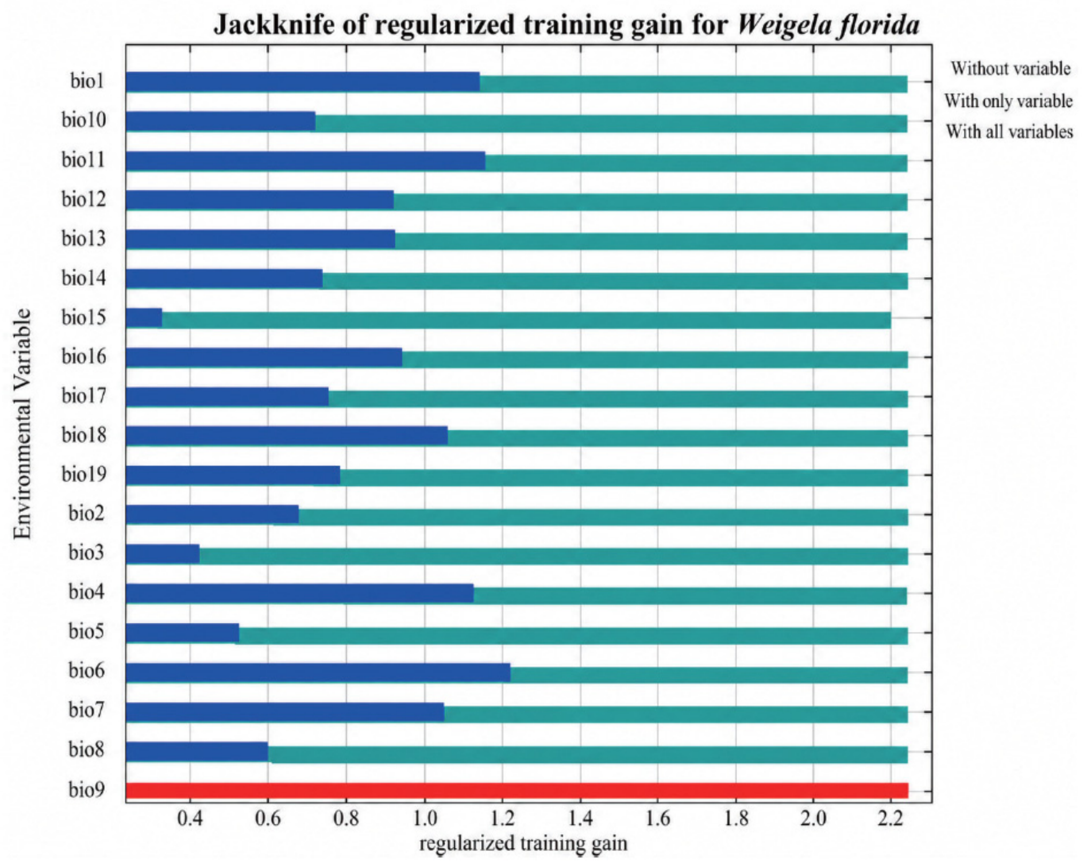

Figure S2. Receiver operating characteristic curve of the MaxEnt model for *Weigela florida*. The red line represents the mean ROC curve, the blue line represents ± one standard deviation, and the black diagonal line represents random prediction. The mean AUC value was 0.966, indicating high model predictive performance.

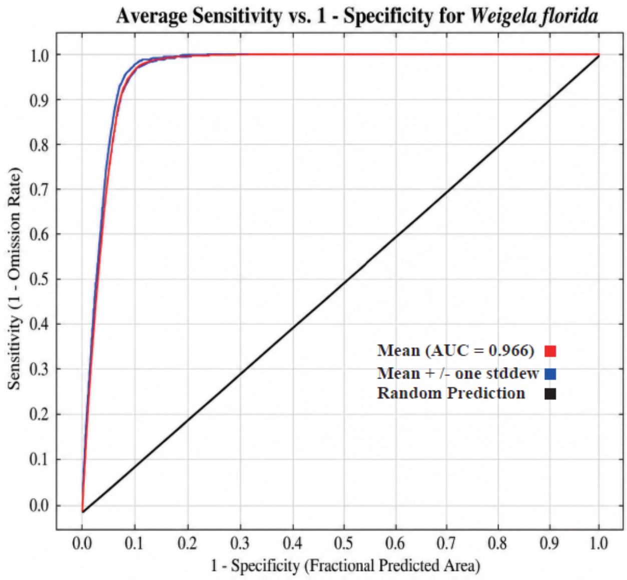

Figure S3. TSS and ROC evaluations of every single model of *Weigela florida*.

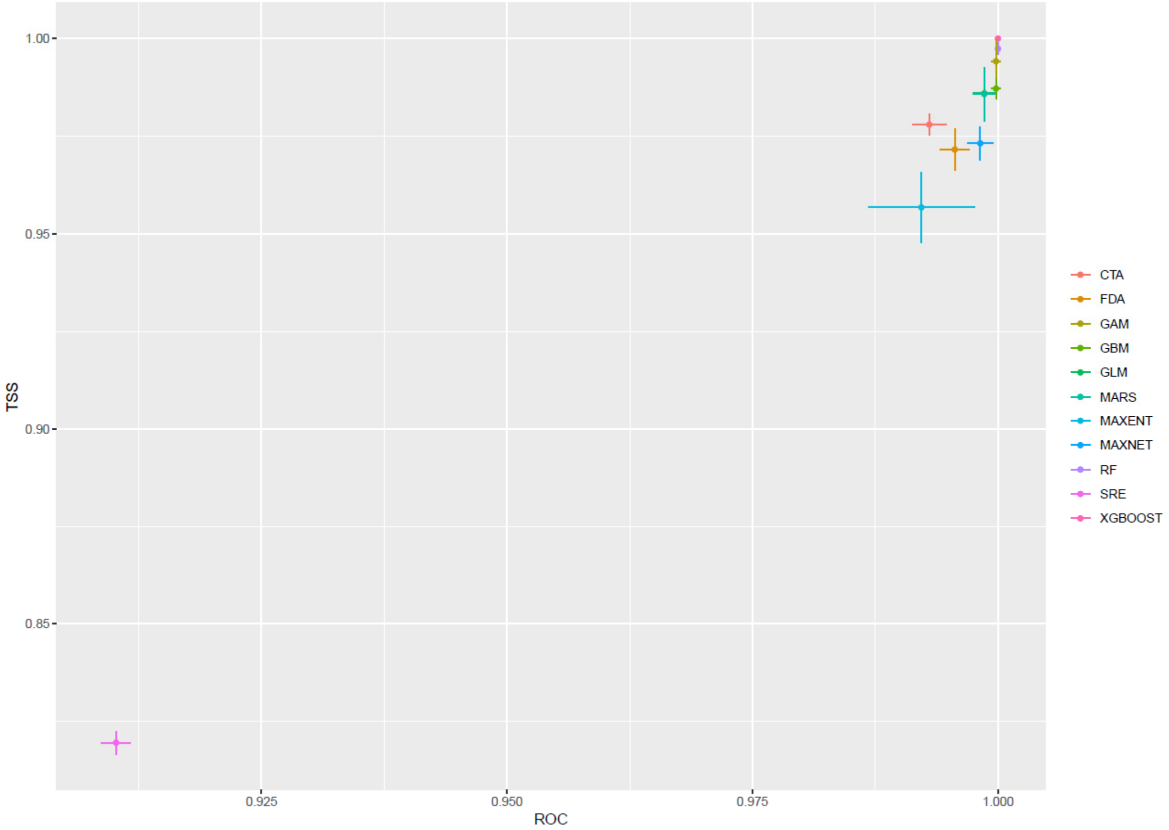

Figure S4. Response curves of *Weigela flor-ida* to three key environmental variables. A: Bio9; B: Bio14; C: Bio18. The red line represents the mean response of 10 replicate MaxEnt runs, and the blue area represents ± one standard deviation.

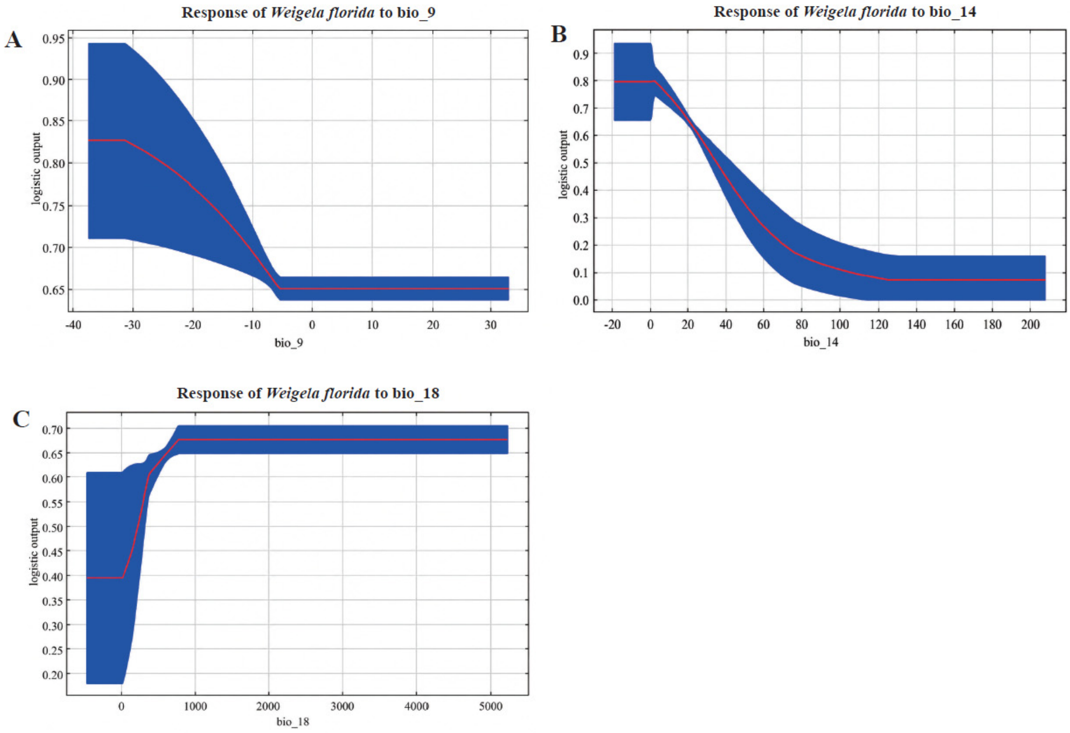

Figure S5. Changes in suitable habitat area of *Weigela florida* under historical and future climate sce-narios. (A) Changes in suitable habitat area from historical periods to the current period. (B–E) Changes in suitable habitat area under SSP126, SSP245, SSP370, and SSP585 scenarios, respectively. The bars represent total suitable area, generally suitable area, moderately suitable area, and highly suitable area.

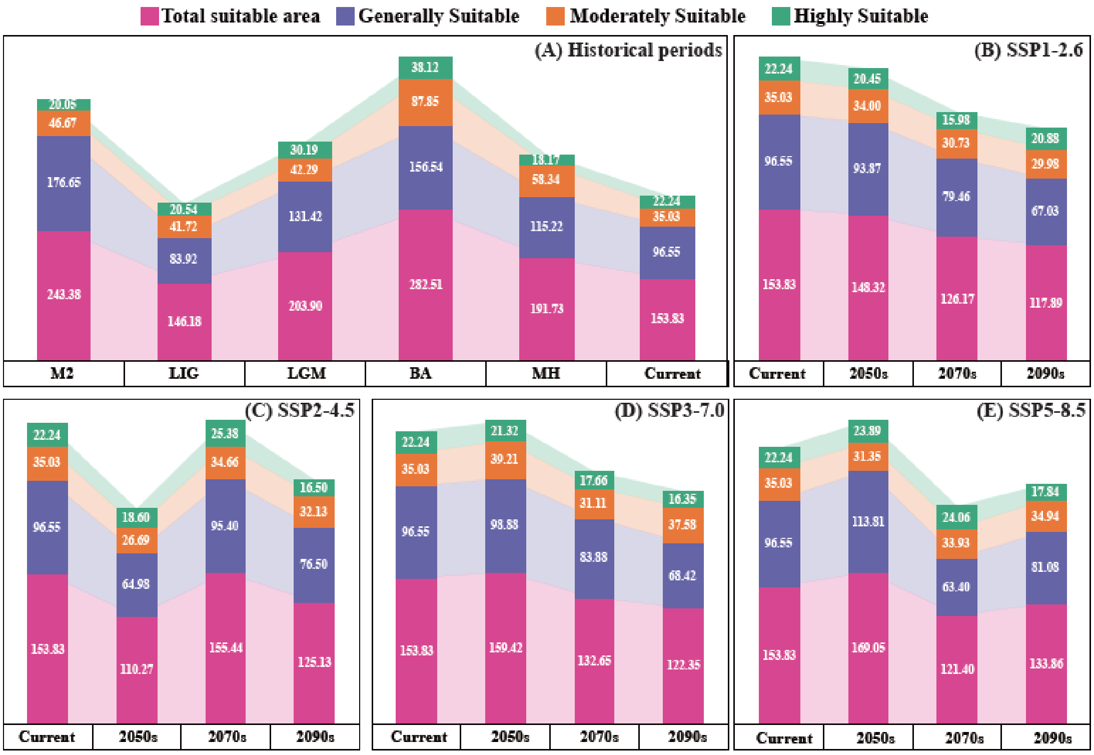

Figure S6. Centroid migration trajectories of suitable habitats of *Weigela florida* under historical and future climate scenarios. (A) Centroid migration trajectory of suitable habitats from historical periods to the current period. (B–E) Centroid migration trajectories from the current pe-riod to future periods under SSP126, SSP245, SSP370, and SSP585 scenarios, respectively. Arrows indicate the direction of centroid migration.

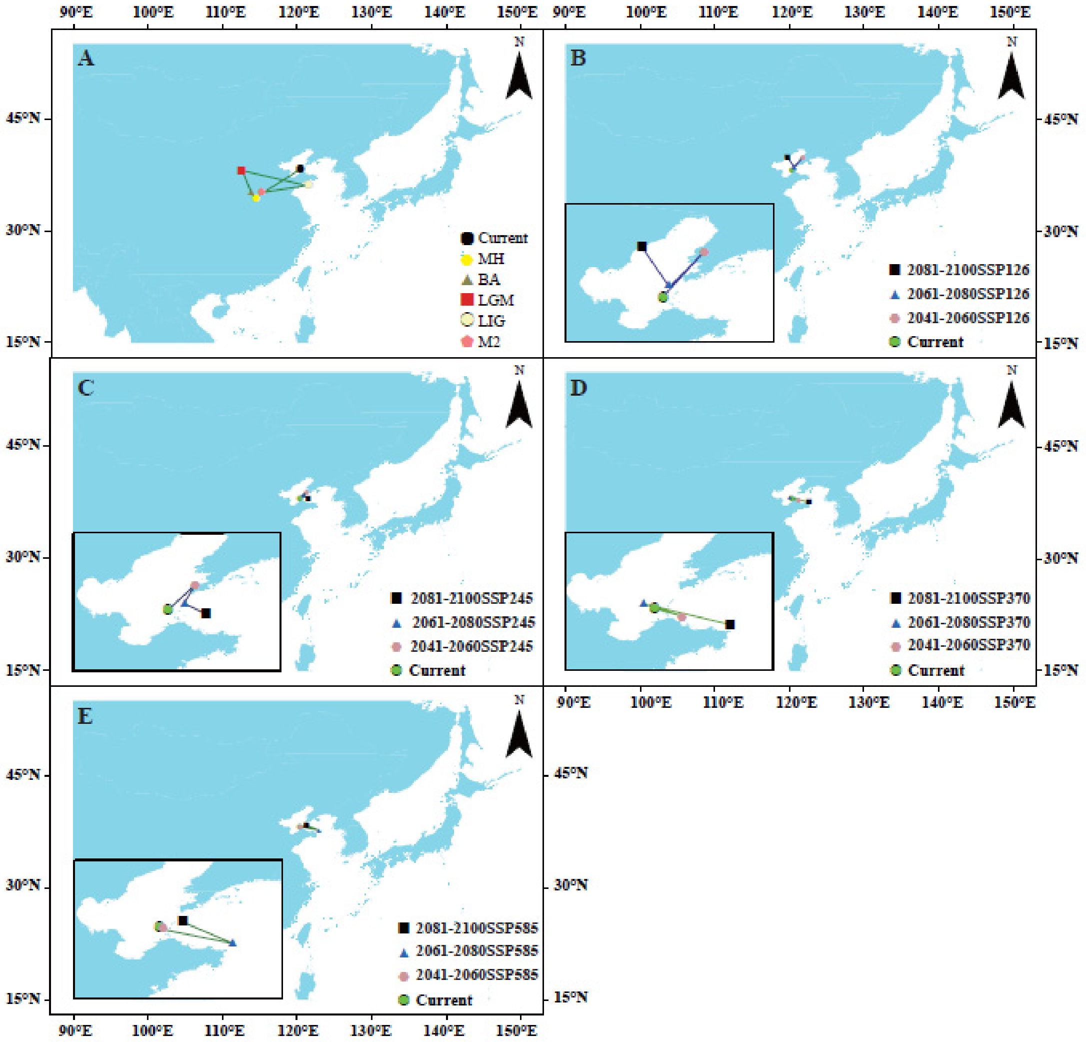

Supplement: Supplementary file 1 [file plants-15-01763-s001.zip › plants-4330760-Figure Supplementary.pdf]
