# Supplementary material for: Spatiotemporal Dynamics of Suitable Habitat for Weigela florida
Source: Plants (Basel). 2026 Jun 7;15(12):1763. doi: 10.3390/plants15121763 (PMC13307421; doi:10.3390/plants15121763)
Supplement: Supplementary file 1 [file plants-15-01763-s001.zip › plants-4330760-Table S1.pdf]

Table S1. List of the 19 bioclimatic variables from the WorldClim database used in this study.

Variables	Description
bio1	Annual Mean Temperature
bio2	Mean Diurnal Range
bio3	Isothermally (BIO2/BIO7) (* 100)
bio4	Temperature Seasonality (standard deviation *100)
bio5	Maximum Temperature of Warmest Month
bio6	Minimum Temperature of Coldest Month
bio7	Temperature Annual Range (Bio5-Bio6)
bio8	Mean Temperature of Wettest Quarter
bio9	Mean Temperature of Driest Quarter
bio10	Mean Temperature of Warmest Quarter
bio11	Mean Temperature of Coldest Quarter
bio12	Annual Precipitation
bio13	Precipitation of Wettest Period
bio14	Precipitation of Driest Period
bio15	Precipitation Seasonality (coefficient of variation)
bio16	Precipitation of Wettest Quarter
bio17	Precipitation of Driest Quarter
bio18	Precipitation of Warmest Quarter
bio19	Precipitation of Coldest Quarter
